# Research for a Common Thread: Insights into the Mechanisms of Six Potential Anticancer Agents

**DOI:** 10.3390/molecules30051031

**Published:** 2025-02-24

**Authors:** Dóra Varga, Anna Szentirmai, András Szarka

**Affiliations:** 1 Laboratory of Biochemistry and Molecular Biology, Department of Applied Biotechnology and Food Science, Budapest University of Technology and Economics, Szent Gellért tér 4, H-1111 Budapest, Hungary; dora.varga@edu.bme.hu (D.V.); anna.szentirmai@edu.bme.hu (A.S.); 2 Biotechnology Model Laboratory, Faculty of Chemical Technology and Biotechnology, Budapest University of Technology and Economics, Szent Gellért tér 4, H-1111 Budapest, Hungary

**Keywords:** reactive oxygen species, cell death, ferroptosis, chloroquine, pharmacologic ascorbate, resveratrol, RSL3, menadione, recombined cancer therapy, drug repurposing

## Abstract

Our research group aimed for the optimization of pharmacologic ascorbate (Ph-Asc)-induced cancer cell death. To reduce the required time and resources needed for development, an in silico system biological approach, an already approved medication, and a mild bioactive compound were used in our previous studies. It was revealed that both Ph-Asc and resveratrol (RES) caused DSBs in the DNA, and chloroquine (CQ) treatment amplified the cytotoxic effect of both Ph-Asc and RES in an autophagy independent way. In the present study, we aimed at the further clarification of the cytotoxic mechanism of Ph-Asc, CQ, and RES by comparing their DNA damaging abilities, effects on the cells’ bioenergetic status, ROS, and lipid ROS generation abilities with those of the three currently investigated compounds (menadione, RSL3, H_2_O_2_). It could be assessed that the induction of DSBs is certainly a common point of their mechanism of action; furthermore, the observed cancer cell death due to the investigated treatments are independent of the bioenergetic status. Contrary to other investigated compounds, the DNA damaging effect of CQ seemed to be ROS independent. Surprisingly, the well-known ferroptosis inducer RSL3 was unable to induce lipid peroxidation in the pancreas ductal adenocarcinoma (PDAC) Mia PaCa-2 cell line. At the same time, it induced DSBs in the DNA, and the RSL3-induced cell death could not be suspended by the well-known ferroptosis inhibitors. All these observations suggest the ferroptosis resistance of this cell line. The observed DNA damaging effect of RSL3 definitely creates a new perspective in anticancer research.

## 1. Introduction

Drug discovery and development is a time and resource consuming process. Therefore, different strategies have been developed to reduce development time and execute appropriate resource management. One of them is drug repurposing, which involves repositioning existing medications to new clinical applications [1]. The bottom line of drug repurposing is the use of an already-approved medication for a new treatment. It often leverages the unexpected (side) effects or unknown therapeutic effects of the medication. By this approach—as the result of bypassing the traditional drug development pathway and transitioning to preclinical and clinical trials—a significant amount of time and money can be saved.

Our research group initiated a pilot project for rational drug development and repurposing. The first step of this rational drug/therapy development was the application of an in silico, systems biology tool [2]. Since the investigation of oxidative stress-driven (cancer) cell death pathways was the main research topic of our laboratory at the beginning of the pilot project, the optimization of pharmacologic ascorbate (Ph-Asc)-induced cancer cell death became the starting point of our project. Pharmacologic ascorbate-induced cancer cell death was an ideal starting point from several points of view. First of all, Ph-Asc treatment is safe, it has been well characterized in clinical safety studies. The intravenous administration elicited no adverse effects or significant physiological/biochemical changes and appeared to be safe up to 100 g [3]. Also an important point at the beginning of our study was that Ph-Asc is commonly applied as a co-therapeutic agent with well-known chemotherapeutics and other bioactive therapeutic agents, such as curcumin [4].

Since KRAS mutations play a key role in tumorigenesis and cancer progression by altering cell signaling, leading to uncontrolled proliferation and resistance to standard therapies, and they can be found in as much as 20–25% of all human tumors [5], our simple in silico model was built, containing the main elements of the regulatory network to provide a detailed qualitative description of the control network in KRAS mutant cancer cells. According to the in silico model, the positive feedback loops of both KRAS and mTOR pathways may have key roles in determining the GLUT1 expression and autophagy induction upon cancer development. The dynamical analysis suggested that the downregulation of KRAS, mTOR, and autophagy could be crucial in anticancer therapy [2]. Chloroquine (CQ) was involved in the model as an autophagy inhibitor [6,7]. The conclusion of the in silico study was that the combined Ph-Asc and CQ treatment was able to block both mTOR and KRAS pathways, which led to the downregulation of both GLUT1 and autophagy [2].

The next step of our pilot project was the investigation of the in vitro effects of the promising combined treatment of Ph-Asc and CQ on pancreas ductal adenocarcinoma (PDAC) cells. Although the previous in silico results could be partially verified, since the combined treatment of Ph-Asc and CQ exerted a synergistic cytotoxic effect on KRAS mutant PDAC cells, it was also found that this effect was not linked to the GLUT1 suppressing effect of Ph-Asc. The autophagy inhibitory effect of CQ also did not play any role in the synergistic cancer cytotoxic effect of Ph-Asc and CQ [8]. Furthermore, their cytotoxic effect was independent of the KRAS pathway and the RAS mutational status of the cancer cells. Beyond our previous results, others also reported that the anticancer effect of CQ was independent of its autophagy inhibitory effect. In a comparative study, ATG7-deficient and non-deficient cells showed equal sensitivity to the anti-proliferative effect of CQ. Furthermore, both cell types manifested synergistic growth inhibition when treated with CQ plus the tyrosine kinase inhibitors, erlotinib or sunitinib [9].

As an mTOR inhibitor, the potential synergistic effect of resveratrol (RES) was also investigated previously [8]. The anticancer effect of the RES co-treatment of MIA PaCa-2 cells with Ph-Asc and/or CQ far exceeded our expectations. The synergistic cytotoxic effect of the investigated three molecules practically killed all the cancer cells. Our findings revealed that RES, similar to Ph-Asc, induced the phosphorylation of H2A.X and Chk1 as the signs of DNA damage [8]. The fact that ascorbate and RES are natural compounds without significant harmful effects on normal cells, and CQ is a known antimalarial drug that can easily be repurposed, gives these observations high importance.

In this study, we aimed for the further clarification of the cytotoxic mechanism of Ph-Asc, CQ, and RES. The observations that both Ph-Asc and RES caused DSBs in the DNA and CQ treatment amplified the cytotoxic effect of Ph-Asc in an autophagy-independent way brought forth the idea that the induction of DSBs can be the mysterious common point that leads to the death of cancer cells. Therefore, three further compounds with oxidative stress-generating capabilities were chosen to confirm this hypothesis. Menadione (vitamin K3) was chosen because of its similarity to Ph-Asc. Both compounds are well-known vitamins (vitamin C and a synthetic form of vitamin K). Although menadione is a synthetic form of vitamin K, it is also formed in the body as the result of a metabolic conversion of the naturally occurring phylloquinone [10]. Similarly to ascorbate menadione, it can participate in redox reactions and generate reactive oxygen species (ROS), which then leads to damage to the DNA. It was observed that the number of DNA breaks is directly proportional to the concentration of menadione and is directly related to the level of ROS [11]. RSL3, as the inhibitor of the lipid peroxide scavenger glutathione peroxidase 4 enzyme (GPX4), triggers the lipid peroxidation-induced cell death, ferroptosis [12,13]). Although it induces an oxidative stress-driven cell death, its effect on the DNA and its DSB forming capability have not been investigated so far. Since the DNA-damaging effect of Ph-Asc is linked to the Fenton reaction, the effect of pure H_2_O_2_ on DSB formation was also investigated. The planned comparison of the pattern of the effects of the three previously investigated compounds (Ph-Asc, resveratrol, CQ) and the three currently being investigated (menadione, RSL3, H_2_O_2_) on DNA damage, the bioenergetic status of the cells, ROS, and lipid ROS generation, and the cell cycle can bring us closer to understanding their mechanisms of action.

## 2. Results

In this study, we aimed to investigate the mechanisms behind the previously observed synergistic effect of Ph-Asc, RES, and CQ [8]. For this purpose, the similarities and differences in the cytotoxic effects of Ph-Asc, RES, and CQ were examined, and as we aimed to further investigate the mechanism behind the cytotoxic effects of Ph-Asc, RES, and CQ, we wanted to compare their cytotoxic mechanisms with other potential anticancer agents known to cause oxidative stress and/or DNA damage, such as the ferroptosis-inducer RSL3, which causes cell death through extensive lipid peroxidation [14,15,16], and menadione, which can cause oxidative stress and DNA damage [17,18,19].

### 2.1. The Disruption of Bioenergetics After DNA Damage Is Only a Secondary Factor in the Toxicity of Ph-Asc and Did Not Occur in the Cell Death Caused by CQ or RES in MIA PaCa-2 Cells

Poly(ADP-ribose) polymerase 1 (PARP1) in response to mild or moderate levels of DNA damage promotes repair mechanisms and cell survival. In contrast, if severe DNA damage occurs, PARP1 activation can lead to the extensive consumption of NAD and ATP and trigger cell death [20,21].

**Figure 1:**
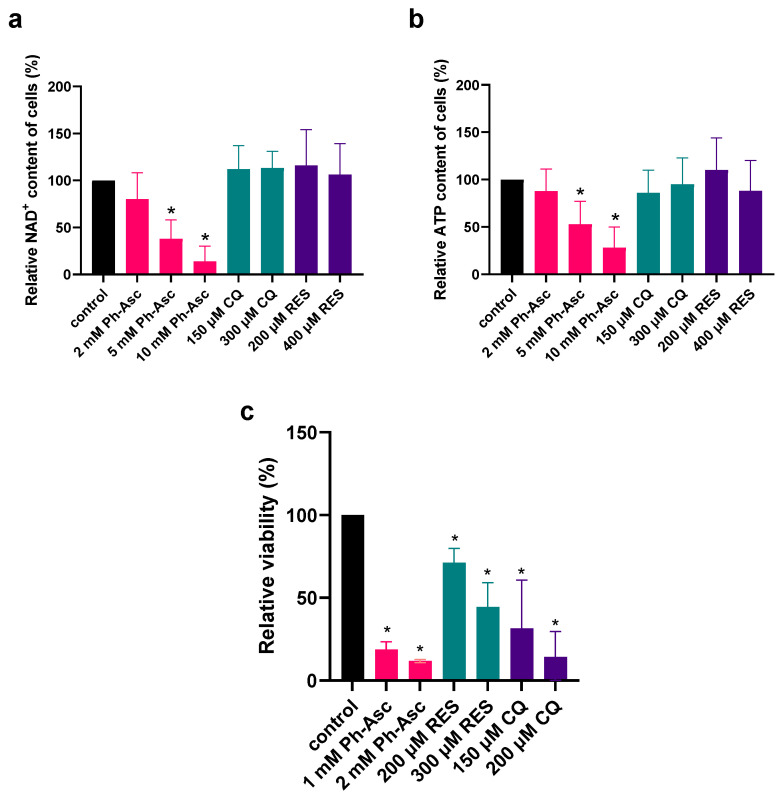
Effect of Ph-Asc, CQ, and RES treatment on bioenergetics of MIA PaCa-2 cells. MIA PaCa-2 cells were cultured on 6 well plates for 24 h and then treated for 2 h with the indicated concentrations and compounds (2–10 mM Ph-Asc, 150–300 µM CQ, 200–400 µM RES). NAD^+^ (**a**) and ATP (**b**) concentrations were measured by HPLC-UV/Vis method and normalized to cell viability as described below. MIA PaCa-2 cells were cultured on 96 well plates for 24 h and then treated with the indicated compounds and concentrations (1 mM and 2 mM Ph-Asc, 150 µM and 200 µM CQ, and 200 µM and 300 µM RES). Cell viability was measured by the MTT assay as described below (**c**). Data are normalized to untreated control and each data point represents the average ± SD from at least 3 independent experiments. * Significantly different (*p* < 0.05) from untreated control.

Buranasudja et al. (2019) found that Ph-Asc-induced DNA damage leads to the activation of PARP1, resulting in intensive NAD^+^ consumption and the consequent depletion of ATP, but indicated that the disruption of bioenergetics is a secondary factor in the toxicity of Ph-Asc, and damage to the DNA appears to be the primary factor [22]. We confirmed that Ph-Asc treatment indeed caused a decrease in the cellular level of NAD^+^ and ATP in MIA PaCa-2 cells, but only in irrelevantly high concentrations. Both the NAD^+^ and ATP content of the cells depleted due to 5 and 10 mM of Ph-Asc treatment, but not due to 2 mM of Ph-Asc treatment, which is sufficient to cause almost complete death of the applied cell line (Figure 1), further confirming that the disruption of bioenergetics does not cause the cell death. Although the disruption of bioenergetics was not the primary cause of cell death in the case of Ph-Asc, we wanted to investigate whether CQ and RES could also cause a similar depletion in the NAD^+^ and ATP content of the cells, as they also caused DNA damage. Despite the presence of DNA damage, no changes in the NAD^+^ and ATP content of the cells could be detected due to CQ and RES treatments, even at concentrations much higher than those sufficient to cause cell death (Figure 1), showing further differences between the mechanisms of action of Ph-Asc, RES, and CQ.

Not surprisingly, Olaparib (a PARP1 inhibitor) treatment could not alleviate the cytotoxic effect of Ph-Asc and also did not affect cell viability after CQ or RES treatment (Figure S1).

### 2.2. Is DNA Damage the Mysterious Common Point of Therapeutics That Leads to the Death of Cancer?

**Figure 2:**
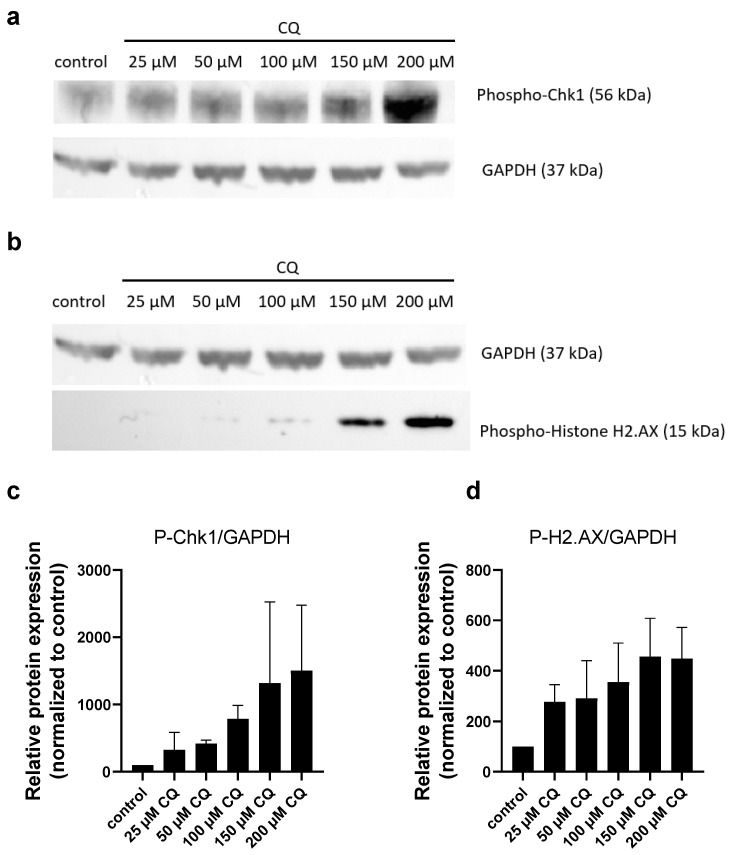
Analysis of DNA damage in MIA PaCa-2 cells in response to CQ treatment: Western blot analysis of total protein samples for Chk1 and Histone H2.AX phosphorylation. MIA PaCa-2 cells were cultured on 6 well plates for 24 h and then treated for 24 h with the indicated concentrations of CQ. Total protein samples were isolated and analyzed as described below. GAPDH were labeled for loading control. One representative Western blot photo of at least three experiments is shown (**a**,**b**). Densitometry data represent the intensity of phosphorylated Chk1 (**c**) and phosphorylated Histone H2.AX (**d**) normalized to GAPDH normalized to untreated control, each data point represents the average ± SD from at least 3 independent experiments.

Previously we found that both Ph-Asc and RES caused DSBs in the DNA [8]. So far, this was the only similarity between the cytotoxic effects of Ph-Asc and RES [8]. It was suspected that DNA damage may be behind the synergistic effect of Ph-Asc, RES, and CQ. Supporting our hypothesis is an increase in the phosphorylation of Chk1 and H2A.X, as markers for DNA damage could be detected in a concentration-dependent manner upon CQ treatment in MIA PaCa-2 cells (Figure 2). This observation further confirms our assumption that DNA damage can be behind the previously mentioned synergistic effect.

As it is generally accepted that the Fenton reaction is in the background of the DNA-damaging effect of Ph-Asc [23], the effect of the pure inorganic Fenton reagent (H_2_O_2_ + FeSO_4_) was compared with Ph-Asc-induced cell death. MIA PaCa-2 cells were treated with a Fenton reagent (50–500 µM H_2_O_2_ + 100–1000 µM FeSO_4_). No difference was found in the cell viability between pure H_2_O_2_ treatment and the combined treatment of H_2_O_2_ and FeSO_4_ (Figure S2), even with FeSO_4_ pre-treatment [24,25]. Based on this observation, it was assumed that the amount of iron present in the culture medium (0.25 μM) was sufficient for the Fenton reaction to occur; therefore, pure H_2_O_2_ treatment was used in the further experiments.

**Figure 3:**
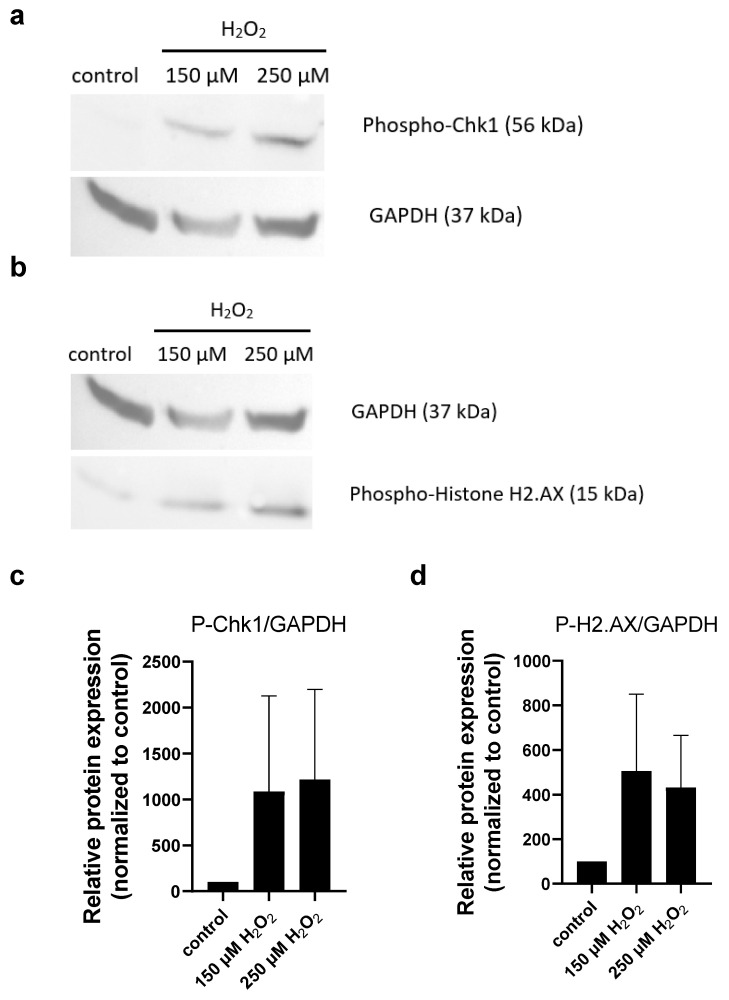
Analysis of DNA damage in MIA PaCa-2 cells in response to H_2_O_2_ treatment: Western blot analysis of total protein samples for Chk1 and Histone H2.AX phosphorylation. MIA PaCa-2 cells were cultured on 6 well plates for 24 h and then treated for 24 h with the indicated concentrations of H_2_O_2_. Total protein samples were isolated and analyzed as described below. GAPDH were labeled for loading control. One representative Western blot photo of at least three experiments is shown (**a**,**b**). Densitometry data represent the intensity of phosphorylated Chk1 (**c**) and phosphorylated Histone H2.AX (**d**) normalized to GAPDH normalized to untreated control, each data point represents the average ± SD from at least 3 independent experiments.

An increase in the phosphorylation of Chk1 and H2A.X proteins could be detected due to H_2_O_2_ treatment in MIA PaCa-2 cells (Figure 3), indicating that H_2_O_2_ caused DNA damage, similar to the Ph-Asc treatment [8].

**Figure 4:**
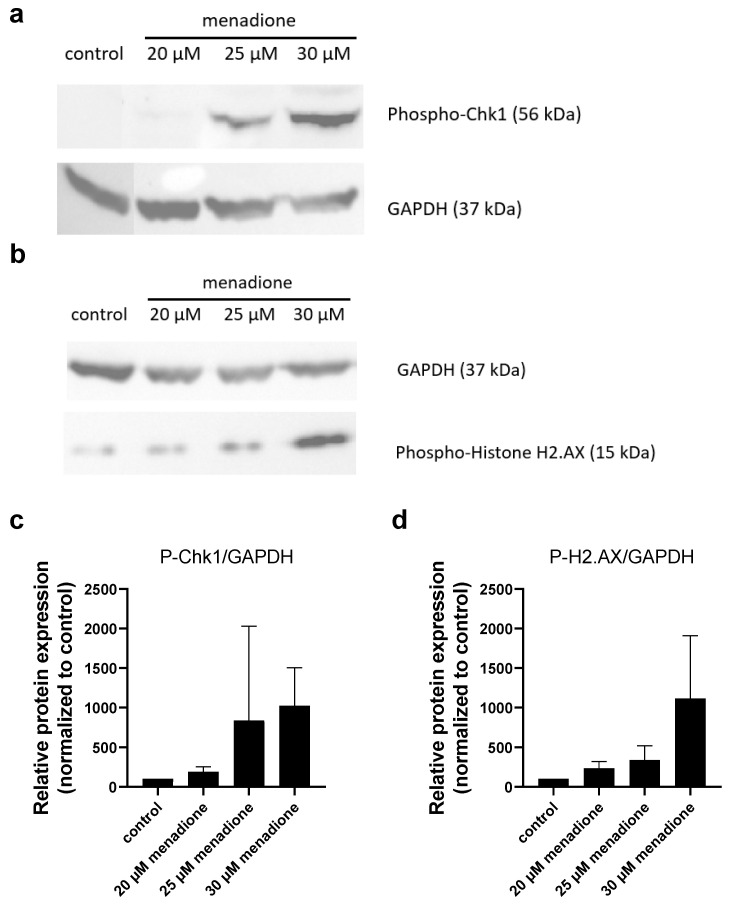
Analysis of DNA damage in MIA PaCa-2 cells in response to menadione treatment: Western blot analysis of total protein samples for Chk1 and Histone H2.AX phosphorylation. MIA PaCa-2 cells were cultured on 6 well plates for 24 h and then treated for 24 h with the indicated concentrations of menadione. Total protein samples were isolated and analyzed as described below. GAPDH were labeled for loading control. One representative Western blot photo of at least three experiments is shown (**a**,**b**). Densitometry data represent the intensity of phosphorylated Chk1 (**c**) and phosphorylated Histone H2.AX (**d**) normalized to GAPDH normalized to untreated control, each data point represents the average ± SD from at least 3 independent experiments.

**Figure 5:**
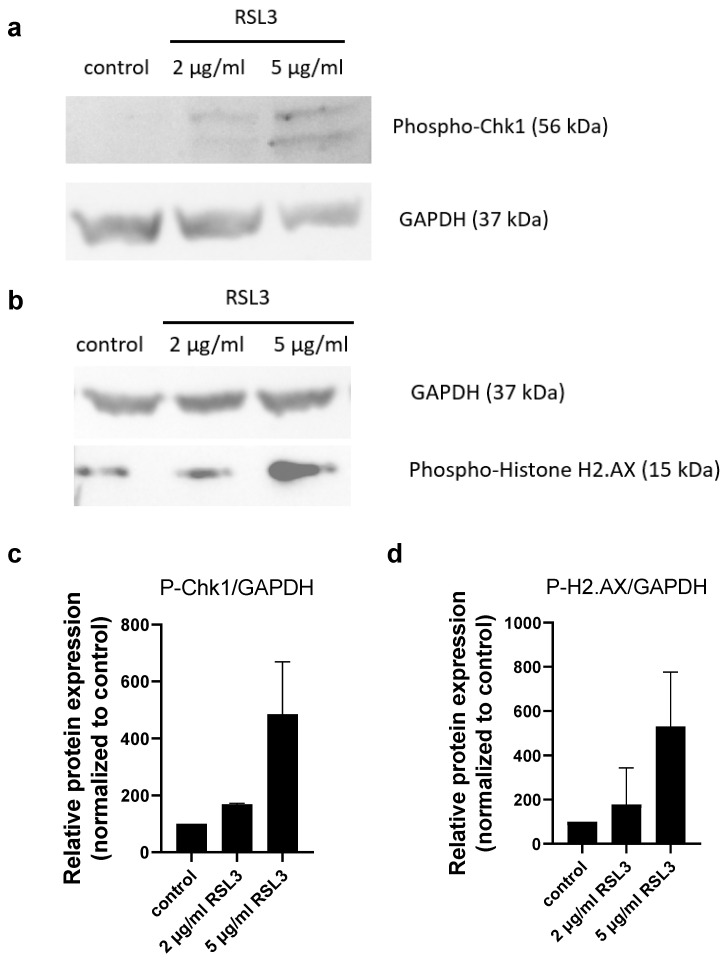
Analysis of DNA damage in MIA PaCa-2 cells in response to RSL3 treatment: Western blot analysis of total protein samples for Chk1 and Histone H2.AX phosphorylation. MIA PaCa-2 cells were cultured on 6 well plates for 24 h and then treated for 24 h with 5 µg/mL RSL3. Total protein samples were isolated and analyzed as described below. GAPDH were labeled for loading control. One representative Western blot photo of at least three experiments is shown (**a**,**b**). Densitometry data represent the intensity of phosphorylated Chk1 (**c**) and phosphorylated Histone H2.AX (**d**) normalized to GAPDH normalized to untreated control, each data point represents the average ± SD from at least 3 independent experiments.

An increase in the phosphorylation of Chk1 and H2A.X proteins could also be detected due to menadione (Figure 4) and RSL3 (Figure 5) treatments in MIA PaCa-2 cells, indicating that both cause DNA damage, similar to all the other potential anticancer agents (Ph-Asc, CQ, RES, H_2_O_2_) investigated in the previous and the present study.

### 2.3. The Effect of Ph-Asc, H_2_O_2_, RES, CQ, Menadione and RSL Treatment on the Cell Cycle

**Figure 6:**
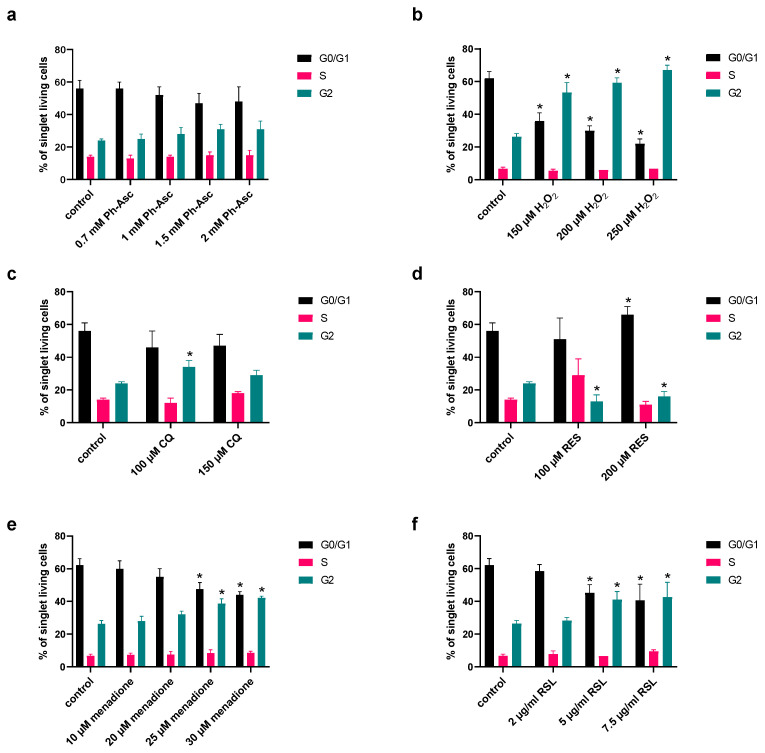
Changes in cell cycle distribution upon Ph-Asc (**a**), H_2_O_2_ (**b**), CQ (**c**), RES (**d**), menadione (**e**), and RSL3 (**f**) treatment on MIA PaCa-2 cells. MIA PaCa-2 cells were cultured on 6 well plates for 24 h and then treated for 24 h with the indicated concentrations and compounds (0.7–2 mM Ph-Asc, 150–250 µM H_2_O_2_, 100–150 µM CQ, 100–200 µM RES, 10–30 µM menadione, 2–7.5 µg/mL RSL3) followed by staining with FxCycle™ PI/RNase Staining Solution, and cell cycle distribution was analyzed by flow cytometry as described below. Cell population was gated using SSC-FSC for singlet fixed cells. Each data point represents the average ± SD of at least three independent experiments. *: Significantly different (*p* < 0.05) from the same cell cycle phase of the untreated control.

All the compounds investigated caused DNA damage, which often leads to cell cycle arrest; therefore, the cell cycle distribution of MIA PaCa-2 cells was analyzed due to Ph-Asc, H_2_O_2_, RES, CQ, menadione, and RSL treatment (Figure 6).

Although the cytotoxic effect of Ph-Asc is attributed to the generation of H_2_O_2_, a difference was found between the mechanisms of the two compounds in their effect on the cell cycle. While Ph-Asc treatment had little or no effect on the cell cycle phase distribution of Mia PaCa-2 cells (Figure 6a), H_2_O_2_ treatment caused a significant cell cycle arrest in a concentration-dependent manner: the proportion of the G0/G1 population was decreased and the proportion of the G2 population was increased (Figure 6b).

Menadione (Figure 6e), RSL3 (Figure 6f), CQ (Figure 6c), and RES (Figure 6d) all caused cell cycle arrest. RES treatment increased the proportion of the G0/G1 population and decreased the proportion of the G2 population, meanwhile CQ, menadione, RSL3, and H_2_O_2_ treatments decreased the proportion of the G0/G1 population and increased the proportion of the G2 population.

### 2.4. The Oxidative Aspects of Ph-Asc, H_2_O_2_, RES, CQ, Menadione and RSL Treatments

As oxidative stress often causes significant damage to macromolecules, including the DNA, the oxidative aspects of Ph-Asc, H_2_O_2_, RES, CQ, menadione, and RSL treatments were analyzed.

**Figure 7:**
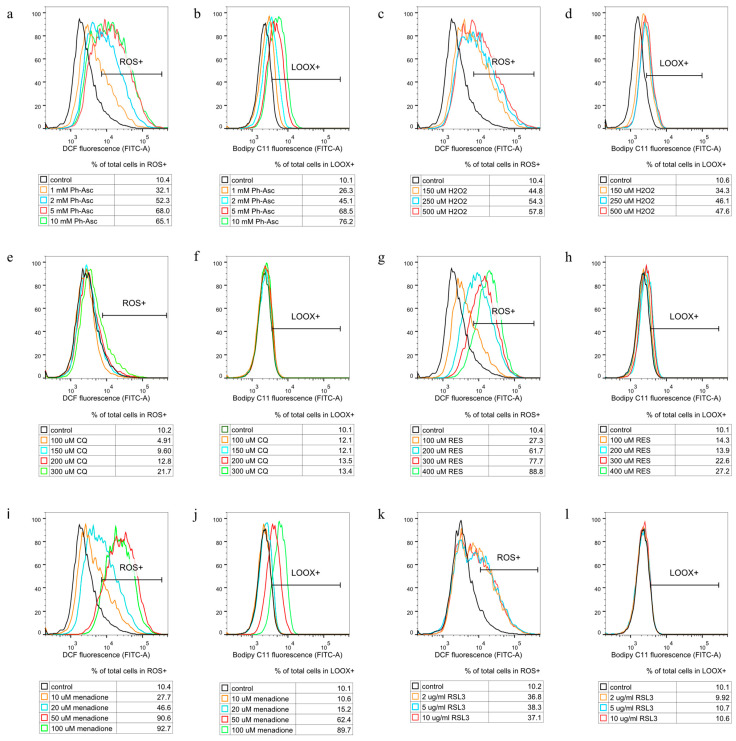
The oxidative aspects of Ph-Asc, H_2_O_2_, CQ, RES, menadione, and RSL3 induced cell deaths in MIA PaCa-2 cells. MIA PaCa-2 cells were cultured on 24 well plates for 24 h, pre-treated with the indicated fluorescent probe (H2DCFDA Invitrogen™ for ROS and BODIPY™ 581/591 C11 for lipid peroxidation measurement) for 30 min, and then treated for 1 h with the indicated concentrations and compounds (1–10 mM Ph-Asc (**a**,**b**), 150–500 µM H_2_O_2_ (**c**,**d**), 100–300 µM CQ (**e**,**f**), 100–400 µM RES (**g**,**h**), 10–100 µM menadione (**i**,**j**) and 2–10 µg/mL RSL3 (**k**,**l**)). Cells were prepared, stained, and measured with a Flow Cytometer as described below. Control samples were fluorescent labeled but untreated. One representative of at least three experiments is shown. Cell population was gated using SSC-FSC and propidium iodide negative staining for singlet living cells.

**Figure 8:**
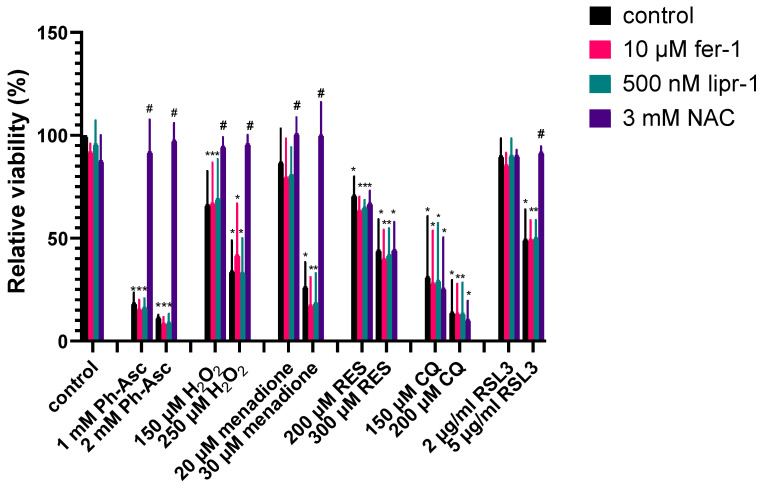
The potential effect of ferroptosis inhibitors and antioxidants in alleviating cell death induced by Ph-Asc, H_2_O_2_, RSL3, CQ, RES, and menadione in MIA PaCa-2 cells. MIA PaCa-2 cells were cultured on 96 well plates for 24 h and then treated with the indicated compounds and concentrations (1 mM and 2 mM Ph-Asc, 150 µM and 250 µM H_2_O_2_, 2 µg/mL and 5 µg/mL RSL3, 150 µM and 200 µM CQ, 200 µM and 300 µM RES, and 20 µM and 30 µM menadione) in the presence or absence of 10 µM ferrostatin-1 (fer-1), 500 nM liproxstatin-1 (lipr-1), and 3 mM n-acetyl-cysteine (NAC). Cell viability was measured by the MTT assay as described below. Data are normalized to untreated control and each data point represents the average ± SD from at least 3 independent experiments. * Significantly different (*p* < 0.05) from untreated control and # significantly different (*p* < 0.05) from group control (same compound and concentration without ferroptosis inhibitor or antioxidant co-treatment).

CQ treatment did not cause any change in the ROS and lipid peroxidation levels of the cell (Figure 7e,f), and its cytotoxic effect could not be alleviated by the antioxidant, N-acetyl-cysteine (NAC) co-treatment (Figure 8), suggesting that ROS formation does not play a key role in CQ-induced DNA damage and cell death.

RES treatment caused elevated ROS levels (Figure 7g) (but not caused lipid peroxidation (Figure 7h)), and its cytotoxic effect could not be alleviated by NAC co-treatment (Figure 8), suggesting that ROS formation, although present, does not play a key role in RES-induced DNA damage and cell death.

Ph-Asc (Figure 7a,b), H_2_O_2_ (Figure 7c,d), and menadione (Figure 7i,j) treatments all resulted in elevated ROS levels and an increase in lipid peroxidation, but H_2_O_2_ treatment induced a much lower degree of lipid peroxidation than Ph-Asc and did not appear to be concentration-dependent. This was found to be another difference between the mechanism of action of Ph-Asc and H_2_O_2_, despite the fact that the cytotoxic effect of Ph-Asc was generally attributed to the generation of H_2_O_2_. While elevated ROS levels and lipid peroxidation are classic features of ferroptosis, it was previously shown that ferroptosis was not involved in Ph-Asc-induced cell death [26]. This was also the case with H_2_O_2_ and menadione, since Ph-Asc, H_2_O_2_, or menadione-induced cell deaths could not be suspended with specific inhibitors of ferroptosis, such as liproxstatin-1 or ferrostatin-1 (Figure 8). However, their effects could be alleviated with the antioxidant NAC co-treatment (Figure 8), confirming that ROS formation has a primary role in the cell death mechanisms due to Ph-Asc, H_2_O_2_, or menadione treatments.

RSL3 treatment elevated the levels of ROS (Figure 7k), but interestingly did not elevate the level of lipid peroxidation (Figure 7l). This was a real surprise since RSL3 is a well-known ferroptosis inducer and lipid peroxidation is one of the characteristic features and inducers of ferroptosis. Based on this, we hypothesized that the MIA PaCa-2 cell line is not sensitive to ferroptosis, which was further confirmed when the known ferroptosis inhibitors, ferrostatin-1 and liproxstatin-1, could not alleviate the cytotoxic effect of RSL3 in MIA PaCa-2 cells, but the cytotoxic effect could be completely suspended with NAC antioxidant co-treatment (Figure 8), suggesting that ROS formation still plays a key role in the cell death.

### 2.5. Menadione Caused Elevated ROS Levels and Lipidperoxidation, but Not Ferroptosis in the Ferroptosis Sensitive HT-1080 Cells

Since menadione is known to induce oxidative stress [17] and it causes lipid peroxidation as well as elevated ROS levels (Figure 7i,j), it was hypothesized that it may also trigger ferroptosis. Since the MIA PaCa-2 cell line was found not to be sensitive to ferroptosis, the HT-1080 cell line was chosen to test the hypothesis that menadione could induce ferroptosis (as ferroptosis was originally described using this cell line [14]).

**Figure 9:**
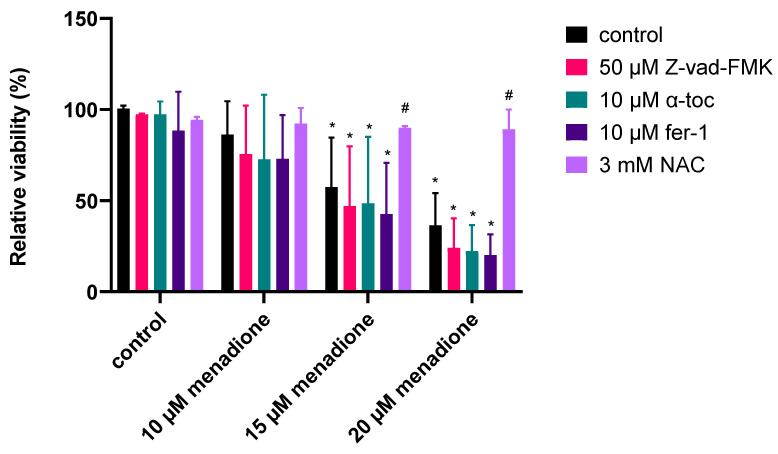
The potential effect of apoptosis inhibition, ferroptosis inhibition, and antioxidant protection in alleviating cell death induced by menadione in HT-1080 cells. HT-1080 cells were cultured on 96 well plates for 24 h and then treated with menadione (5–50 µM) in the presence or absence of 50 µM Z-vad-FMK, 10 µM α-tocopherol (α-toc), and 10 µM ferrostatin-1 (fer-1). Cell viability was measured by the MTT assay, as described below. Data are normalized to untreated control and each data point represents the average ± SD from at least 3 independent experiments. * Significantly different (*p* < 0.05) from untreated control and # significantly different (*p* < 0.05) from group control (same compound and concentration without ferroptosis inhibitor or antioxidant co-treatment).

While menadione elevated ROS levels and lipid peroxidation in the HT-1080 cell line (Figure S3), similar to the MIA PaCa-2 cell line (Figure 7i,j), the specific ferroptosis inhibitor ferrostatin-1 and the antioxidant α-tocopherol (α-toc) could not alleviate the menadione-induced cell death (Figure 9), albeit, it was shown previously that both ferrostatin-1 and α-tocopherol could suspend the ferroptotic cell death induced by RSL3 in HT-1080 cells [16]. Interestingly the pan-caspase inhibitor Z-vad-FMK also could not alleviate the menadione-induced cell death, while the antioxidant NAC could completely suspend the menadione-induced cell death in HT-1080 cells (Figure 9), similar to the MIA PaCa-2 cells (Figure 8), suggesting that while apoptosis does not, ROS formation does play a role in the cell death.

## 3. Discussion

In a recently initiated project, our research group aimed for the optimization of Ph-Asc-induced cancer cell death. To reduce the required time and resources for its development, an in silico system biological approach, an already approved medication, and a mild bioactive compound were used [2,8].

On the basis of the first in silico study, the Ph-Asc and the repurposed anti-malarial agent CQ combined treatment was tested on different KRAS mutant and non-mutant cancer cell lines [2,8]. The results of the in silico study could be partly confirmed, and it was assumed that the cytotoxic effect of Ph-Asc and CQ was not the unique feature of PDAC cell lines harboring KRAS mutation [8]. The in vitro study raised further ideas and questions:

1. Can DNA damage (DSBs) be the common point in the background of the cytotoxic effect of the investigated compounds?
2. Does the bioenergetic rupture of the cells play any role in the investigated DNA damaging agents induced cell death?
3. Is there any difference between cancer cell death induced by Ph-Asc treatment and a pure Fenton reaction?
4. What is the difference between Ph-Asc and other oxidative stress-inducing agents, such as menadione- and RSL3-induced cancer cell death?

According to our previous study, DNA damage was the only common point in the background of the cytotoxic effect of Ph-Asc and resveratrol [8]. As DNA damage is often caused by oxidative stress, three further compounds with oxidative stress-generating capabilities (menadione, RSL3, H_2_O_2_) and the non-oxidative cytotoxic agent CQ were chosen to confirm this assumption.

The concentration-dependent increase in the phosphorylation of two DNA damage marker proteins, Chk1 and H2A.X, could also be observed due to CQ treatment (Figure 2). Our result is not without example since the significant elevation of the oxidative DNA damage marker 8-oxodG was reported by a study investigating the genotoxic potential of CQ in primary mouse embryonic fibroblasts [27]. Furthermore, the synergistic cancer toxic effect of CQ and nonhomologous end joining inhibitors was observed in ovarian cancer cell lines reinforcing the role of DSBs in the mechanism of cancer cell cytotoxicity [28,29]. However, an interesting difference can be observed between the results gained on ovarian cancer cell lines and on MIA PaCa-2 pancreatic cancer cells. While the CQ-induced cell death was found to be of oxidative nature and it can be suspended by NAC addition in ovarian cancer cell lines [28,29], ROS or lipid ROS formation could not be observed on MIA PaCa-2 pancreatic cancer cells due to CQ treatment (Figure 7e,f), and it could not be suspended by NAC addition (Figure 8). The observed difference is quite common in cell lines with different origins [30,31,32]. In summary, these results reinforced our assumption that DNA damage (DSBs) can be the common point in the cytotoxic effect of the investigated compounds.

Ph-Asc induced DNA damage results in the over-activation of PARP1. The over-activation of PARP1 implies the consumption of NAD^+^ and, subsequently, the depletion of ATP leading to mitotic cell death. Although the inhibition or the genetic deletion of PARP1 did not prevent cytotoxicity of Ph-Asc on pancreatic cancer cells, suggesting that it was DNA damage and not the disruption of cellular bioenergetics that was the predominant factor for the anticancer activity of Ph-Asc [22], we aimed to investigate the effect of Ph-Asc and the other two investigated compounds (CQ and resveratrol) on the bioenergetics status (cellular level of NAD^+^ and ATP) of pancreatic cancer cells. Both the concentration of Ph-Asc that induced the depletion of the cellular NAD^+^ and ATP pool and the degree of the depletion was very similar to what was observed earlier (Figure 1, [22]), validating our experimental setup. However, no change could be detected in the NAD^+^ and ATP levels due to chloroquine and resveratrol treatment (Figure 1). At this point, it should also be noted that while the cell death occurred at concentrations of Asc as low as 2 mM, the depletion in NAD^+^ and ATP concentration are initiated only at concentrations as high as 5 mM (Figure 1). All these observations underline the assumption that the observed cancer cell death due to Ph-Asc, resveratrol, and chloroquine treatment are independent of the bioenergetics status of the investigated cell lines.

The cancer-killing effect of Ph-Asc is ascribed to its H_2_O_2_ generating ability and to its role in the maintenance of the Fenton reaction [8,22,23]. We would have liked to clarify whether there was any additional special role of the ascorbate molecule in the cell death of cancer cells or if it was just an H_2_O_2_ generator. Thus, the cancer killing effect of Ph-Asc and the inorganic Fenton reagent was compared. At the beginning, it became clear that the iron content of the cell culture media was enough for the continuous Fenton reaction, hence the experimental setup could be simplified into the comparison of the effect of H_2_O_2_ and Ph-Asc treatments. The main effects of the two treatments were highly similar: (i) both induced the phosphorylation of Chk1 and H2A.X proteins indicating DNA damage (Figure 2, [8,22]); (ii) both induced the elevated generation of ROS and increased lipid peroxidation (Figure 7a–d); (iii) both showed similar inhibitory profile and ferroptosis independence (Figure 8, [26]). Only slight difference could be observed between the two treatments. H_2_O_2_ treatment caused only moderate and non-concentration-dependent lipid peroxidation (Figure 7a–d). Furthermore, Ph-Asc treatment had little or no effect on the cell cycle phase distribution of Mia PaCa-2 cells (Figure 6a, [8]); however, H_2_O_2_ treatment caused a significant cell cycle arrest in a concentration-dependent manner. It decreased the proportion of the G0/G1 population and increased the proportion of the G2 population compared to the untreated control (Figure 6b). The differences both in the lipid peroxidation levels and cell cycle arrest may be the result of the different time interval of exposure of the cells to H_2_O_2_. H_2_O_2_ causes a sudden, large amount of ROS formation in a matter of seconds, but it is an unstable substance that is broken down by several enzymes (catalase, GPXs) and is quickly eliminated after an initial oxidative stress, while Ph-Asc can maintain a lower but continuous level of H_2_O_2_ and oxidative stress by continuously reducing transition metal ions, thus maintaining the Fenton reaction for a longer time.

Since menadione has properties very similar to Ph-Asc, we were curious about the result of menadione treatment on Mia Paca-2 cells, so we included it in our experiment. What are these similar properties? Menadione or vitamin K3 is a product of phylloquinone (vitamin K1) metabolism, it can participate in redox reactions and generate ROS, which then leads to damage to the DNA and other macromolecules. ROS production plays a primal and important role in menadione-mediated DNA damage [10,33]. Menadione could induce the formation of both SSBs and DSBs in MCF-7 breast cancer cells in a ROS and concentration dependent manner [10,18]. Menadione has been shown to induce DNA strand breaks in different cell types [10]. The observed increase in the phosphorylation of Chk1 and H2A.X after menadione treatment confirmed the previous reports and our expectations (Figure 4). As a result of DNA damage menadione—similar to CQ (Figure 6c) and RES (Figure 6d)— cell cycle arrest occurred (Figure 6e).

The observation that menadione-induced lipid peroxidation and elevated ROS levels (Figure 7i,j) brought forth the idea that it may trigger ferroptosis, the lipid peroxidation-driven cell death. The well-known inducer of ferroptosis RSL3 was used as a reference [14]. The results were more than surprising since menadione treatment caused an increase in lipid peroxidation (Figure 7j), but RLS3 treatment did not (Figure 7l). The ineffectiveness of the well-known and generally accepted ferroptosis inhibitors (ferrostatin-1 and liproxstatin-1) to alleviate the RSL3-treated MIA PaCa-2 cells reinforced our assumption that MIA PaCa-2 cells seem to be ferroptosis resistant (Figure 8). According to The Human Protein Atlas database, the MIA PaCa-2 cell line has a much higher GPX4 expression level [34] and much lower ACSL4 expression level [35] than the average of the most common cell lines and the ferroptosis-sensitive HT-1080 cell line, which could be the reason behind its ferroptosis resistance. Since we could not find any publications in Pubmed that applied RSL3 treatment on MIA PaCa-2 cells, it is the first description of the ferroptosis resistance of this cell line. The menadione treatment-induced lipid peroxidation and the ferroptosis resistance of the MIA PaCa-2 cell line drove us to test the ferroptosis-inducing ability of menadione on the HT-1080 cell line, the cell line on which ferroptosis was described [14]. However, similarly to the MIA PaCa-2 cell line, the well-known ferroptosis inhibitor ferrostatin-1 and the antioxidant α-tocopherol could not alleviate the menadione-induced cell death in the case of HT-1080 (Figure 9). Similarly, the pan-caspase inhibitor Z-vad-FMK could not alleviate the menadione-induced cell death, while the antioxidant NAC could completely suspend the menadione-induced cell death in HT-1080 cells (Figure 9). These observations suggest that ROS formation plays an important role in menadione-induced cell death, but ferroptosis and apoptosis are not likely to be involved in it.

At this point, it should be noted that although RSL3 could not induce lipid peroxidation and ferroptosis in MIA PaCa-2 cells, it behaved similar to resveratrol: it could induce elevated ROS formation and damage DNA. It is the first time that the DNA damaging effect of RSL3 has been described.

Based on our experiments, we could answer the four previously addressed questions:

1. Can the DNA damage (DSBs) be the common point in the background of the cytotoxic effect of the investigated compounds?
  Although there are differences in the effects of the six compounds investigated (Ph-Asc, H_2_O_2_, CQ, RES, menadione, RSL3), there is one common point: the damage to the DNA. According to our results, the induction of DSBs can be the common point in the background of their cytotoxic effect.
2. Does the bioenergetic rupture of the cells play any role in the investigated DNA damaging agents induced cell death?
  Our results reinforced the assumption that the observed cancer cell death due to the investigated treatments are independent of the bioenergetics status.
3. Is there any difference between cancer cell death induced by Ph-Asc treatment and a pure Fenton reaction?
  It seems there is only slight difference between the cancer cytotoxic effect of Ph-Asc and the Fenton reagent, which can originate from the different time interval of exposure of the cells to H_2_O_2_ due to the different treatments (Ph-Asc vs. H_2_O_2_).
4. What is the difference between Ph-Asc and another oxidative stress-inducing agent, menadione-induced cancer cell death?
  The treatment of MIA PaCa-2 cells with Ph-Asc or menadione resulted in very similar effects: both induced elevated ROS and lipid ROS generation and DNA damage. It is not accidental that Ph-Asc had a synergistic effect on menadione treatment [36,37,38]. The Ph-Asc and menadione co-treatment lead to a significant increase in oxidative stress and a decrease in the mitochondrial membrane potential, which has a key role in triggering cancer cell death [37,39,40]. These observations again underline the rational of this combination treatment. Combining two or more drugs might lead to increased efficacy, multidirectional cellular activity, or the suppression of drug resistance, thus permitting lower doses [10].

Necessarily, our new results provided new questions: the potential ferroptosis resistance of MIA PaCa-2 and the DNA damaging effect of RSL3 are exciting problems which should be clarified in the near future.

## 4. Materials and Methods

### 4.1. Cell Cultures

MIA PaCa-2 (obtained from ATCC Database, ATCC Number: CCL-121), a human pancreatic cancer cell line, and the HT-1080 (obtained from ECACC Database, Catalogue No.: 85111505) fibrosarcoma cell line used in the experiments were cultured according to ATCC guidelines. Cells were grown in a cell culture incubator (Thermo Scientific™, Waltham, MA, USA, Forma™ Series II 3111) at 37 °C, 5% CO_2_, 100% relative humidity. The complete culture medium for the MIA PaCa-2 cell line comprising high glucose DMEM with stable glutamine (Thermo Scientific™, Gibco™, Waltham, MA, USA, 10566016 or Sigma-Aldrich^®^, St. Louis, MO, USA, D0819) was supplemented with 10% FBS (Sigma-Aldrich^®^, F7524) and 2,5% horse serum (Thermo Scientific™, Gibco™, 16050130). MIA PaCa-2 cells were subcultured routinely before reaching 100% confluence in a ratio of 1:4 or 1:6. The complete culture medium for the HT-1080 cell line comprising high glucose DMEM with stable glutamine (Thermo Scientific™, Gibco™, 10566016 or Sigma-Aldrich^®^, D0819) was supplemented with 10% FBS (Sigma-Aldrich^®^, F7524). HT-1080 cells were subcultured routinely before reaching 100% confluence in a ratio of 1:6 or 1:8.

### 4.2. Treatment of MIA PaCa-2 and HT-1080 Cells for MTT Viability Assay, Flow Cytometry, Protein Isolation, or NAD^+^ and ATP Measurement Sample Preparation

HT-1080 cells were seeded homogenously 24 h prior to treatment in either 96, 24, or 6 well plates (Thermo Scientific™ BioLite) at a seeding density of 1.5 × 10^4^, 1 × 10^5^, 5 × 10^5^ cells/well, respectively, in a complete culture medium. MIA PaCa-2 cells were seeded homogenously 24 h prior to treatment in 96, 24, or 6 well plates (Thermo Scientific™ BioLite), at a seeding density of 3 × 10^4^, 2.5 × 10^5^, 1 × 10^6^ cells/well, respectively, in a complete culture medium. After 24 h of incubation, the complete culture medium was renewed and supplemented with various compounds for treatment: a 0.5–10 mM final concentration of ascorbic acid (Sigma-Aldrich^®^, A7506, solved in complete culture medium, stock solution was prepared before each treatment in complete culture medium and the pH was adjusted to phenol red neutral with sodium hydroxide), 150–500 μM final concentration of H_2_O_2_ (Molar Chemicals Ltd., Halásztelek, Hungary, 7722-84-1), 100–400 μM final concentration of resveratrol (MedChemExpress^®^, MCE^®^, Monmouth Junction, NJ, USA, HY-16561, solved in DMSO), 25–300 μM final concentration of chloroquine (Sigma-Aldrich^®^, C6628, solved in a complete culture medium; the stock solution was prepared before each treatment in a complete culture medium, and the pH was adjusted to phenol red neutral with sodium hydroxide), 10–100 μM final concentration of menadione (Sigma-Aldrich^®^, C6628, solved in DMSO) or 2–10 μg/mL final concentration of RSL3 (MCE^®^, HY-100218A, solved in DMSO). For inhibitor profile studies, the complete culture medium was further supplemented by one of the following: a 50 μM final concentration of Z-VAD-FMK (MCE^®^, HY-16658B, solved in DMSO), 500 nM final concentration of liproxstatin-1 (MCE^®^, HY-12726, solved in DMSO), 10 μM final concentration of ferrostatin-1 (MCE^®^, HY-100579, solved in DMSO), 10 μM final concentration of α-tocopherol (Sigma-Aldrich^®^, 258024, solved in DMSO), 3 mM final concentration of N-acetyl-cysteine (MCE^®^, HY-B0215, solved in complete culture medium; the stock solution was prepared before each treatment in a complete culture medium and the pH was adjusted to phenol red neutral with sodium hydroxide) or a 1–10 μM final concentration of Olaparib (Sigma-Aldrich^®^, SML3705, solved in DMSO). For the Fenton-reagent cells were treated for 24 h with a final concentration of 50–500 µM H_2_O_2_ and 100–1000 µM FeSO_4_ (solved in complete culture medium, the stock solution was prepared before each treatment) or pre-treated with a final concentration of 100–1000 µM FeSO_4_ for 1 h, then washed twice with PBS and then treated with a final concentration of 50–500 µM H_2_O_2_ for 24 h.

Solvent (DMSO, Sigma-Aldrich^®^, D8414) controls were used in all cases for inhibitor profile studies, maximum DMSO content was 0.1 *v*/*v*%).

### 4.3. Measurement of Cell Viability with the MTT Assay

Cell viability for toxicity and inhibitor profile determination was measured in 96 well plates. Cells were seeded and treated as described above. After 24 h of treatment, the medium was discarded, and the plate was washed twice with PBS. To assess cell viability, the complete culture medium supplemented with 1/10 volume 5 mg/mL MTT (Sigma-Aldrich^®^, M2003, dissolved in PBS) was added to the plate and incubated for 40 min until sufficient formazan crystals were present. After incubation, the medium was discarded, and the formazan crystals were dissolved by adding DMSO and incubating the plate for 10 min at 37 °C. The absorbance of the formazan solution was determined by a microplate spectrophotometer (Thermo Scientific™ Multiskan™ GO) at 570 nm using the absorbance at 690 nm as a background. All viability values were normalized to an untreated control on the same plate.

### 4.4. Measurement of ROS and Lipid Peroxidation Using Flow Cytometry

Cells were seeded and treated as described above in 24 or 6 well plates, then after 24 h of incubation, the cells were pre-treated with the fluorescent probes at the following final concentrations: 20 µM dichlorofluorescein-diacetate (DCF-DA, Thermo Scientific™, D399), 2 µM BODIPY™ 581/591 C11 (Bodipy-C11, Thermo Scientific™, D3861) for 30 min in 37 °C; after the pre-treatment, the culture medium was discarded, and the cells were washed twice with PBS and treated as described above. After the indicated treatment and time, the culture medium was discarded, and the cells were washed twice with PBS, trypsinised, and resuspended in the complete culture medium. The cells were analyzed in a CytoFLEX™ S (Beckman Coulter™, Brea, CA, USA) Flow Cytometer. The cell population was focused with unstained cells and all dyes were used separately to avoid interference. A minimum of 10,000 cells were analyzed per condition. Data were analyzed using FlowJo^®^ (version: 10.0.7r2), and the cell population was gated using unlabeled cells. Histograms are normalized to the samples’ mode.

### 4.5. Measurement of Cell Cycle Phase Distribution Using Flow Cytometry

Cells were treated on 6-well plates as described above. After 24 h of treatment, the culture medium was discarded, and the cells were washed twice with PBS, trypsinised, and resuspended in culture medium. An amount of 100 µL from the cell suspension was used for the determination of viable cell number using the CytoFLEX™ (Beckman Coulter™) Flow Cytometer. An amount of 500 µL PBS was added to a suspension of 100,000 cells, and then cells were centrifuged at 500× *g* for 3 min. The samples were fixed in 70% ethanol on ice for 30 min, and then fixed cells were centrifuged again at 1000× *g* for 3 min. The ethanol was discarded, and the cells were resuspended in 150 µL FxCycle™ PI/RNase Staining Solution (Thermo Scientific ™, F10797) and incubated for 15 min in room temperature, protected from light. Cell cycles were analyzed based on DNA content using an Attune™ NxT, Acoustic Focusing Flow Cytometer (Thermo Scientific™, model: AFC2) or CytoFLEX™ (Beckman Coulter™) Flow Cytometer, and the emission of fluorescent dyes was measured on the 574/26 nm channel. A minimum of 10,000 cells were analyzed per condition.

### 4.6. Measurement of NAD^+^ and ATP Using HPLC-UV/Vis Methods

Cells were seeded in 6 well plates and treated as described above. After 24 h of treatment, the culture medium was discarded, and the cells were washed twice with PBS, trypsinised, and resuspended in culture medium. An amount of 100 µL from the cell suspension was used for the determination of a viable cell number using the CytoFLEX™ (Beckman Coulter™) Flow Cytometer. Cells were centrifuged at 500× *g* for 3 min, the supernatant was discarded, and cells were resuspended in 150 μL 5% sulfosalicylic acid (SSA) and then incubated on ice for 15 min; after the 15 min, the cells were centrifuged at 21,200× *g* for 5 min at 4 °C. The supernatant was neutralized with 50 μL 3 M K_2_CO_3_ solution and stored at −80 °C until analysis.

NAD^+^ analysis was based on a method from Yoshino J. and Imai S. [41]. For separation, a Waters Acquity UPLC H-Class system (Waters, Milford, MA, USA) was used, equipped with an Acquity UPLC BEH C18 2.1 × 50 mm column with an average particle diameter of 1.7 μm. Gradient elution was used as 50 mM of potassium hydrogen phosphate (pH 7.0) and methanol. The detector was a Waters Acquity PDA detector, and absorbance was monitored at 261 nm. Quantitation was achieved by measuring NAD^+^ standards (Reanal Laboratory Chemicals Ltd., Budapest, Hungary, 53-84-9).

ATP analysis was based on a method from Juarez-Facio A. T. et al. [42]. For separation, a Waters Acquity UPLC H-Class system was used, equipped with an OOB-4760-YO Luna^®^ Omega Polar C18 100 Å 50 × 3.0 mm column (Phenomenex^®^, Torrance, CA, USA) with an average particle diameter of 3 μm, using isocratic elution with a mobile phase consisting of 50 mM of potassium hydrogen phosphate (pH 6.80). The detector was a Waters^®^ Acquity PDA detector, and absorbance was monitored at 254 nm. Quantitation was achieved by measuring ATP standards (Sigma-Aldrich^®^, A2383).

### 4.7. Isolation and Quantitation of Protein Samples

Cells were seeded in 6 well plates and treated as described above. After 24 h of treatment, the cells were lysed in RIPA protein isolation buffer (150 mM NaCl, 1% NP-40, 50 mM Tris pH 8.0) supplemented with 1% protease inhibitor cocktail (Sigma-Aldrich^®^, P8340), 1% phosphatase inhibitor cocktail (Sigma-Aldrich^®^, P5726), and 1 mM PMSF (Sigma-Aldrich^®^, 78830). Samples were incubated on ice for 30 min, then centrifuged at 14,000× *g* for 15 min at 4 °C. The supernatant was stored at −80 °C until protein analysis.

Protein samples were quantified using the Pierce™ BCA Protein Assay Kit (Thermo Scientific™, 23225) according to the manufacturer’s guidelines.

### 4.8. Analysis of Protein Samples Using Western Blot

SDS-PAGE was performed by using Mini Gel Tank (Thermo Scientific™, A25977) with NuPAGE™ Bis-Tris Mini Protein Gels (Thermo Scientific™, NP0336, NP0302) and NuPAGE™ MOPS SDS Running Buffer (Thermo Scientific™, NP0001). Proteins were transferred onto the Millipore 0.45 µM nitrocellulose membrane by using the Power Blotter System (Thermo Scientific™, PB0013) and the Power Blotter 1-Step™ Transfer Buffer (Thermo Scientific™, PB7100). Immunoblotting was performed using TBS Tween (0.1%), containing 5% non-fat dry milk for a blocking membrane and 1% non-fat dry milk for the antibody solutions. Loading was controlled by developing membranes for GAPDH in each experiment.

The following antibodies were applied: Phospho-Chk1 (Ser345) (133D3) Rabbit mAb (Cell Signaling Technology^®^, CST^®^, Danvers, MA, USA, 2348, dilution: 1:250), Phospho-Histone H2A.X (Ser139) (20E3) Rabbit mAb (CST^®^, 9718, dilution: 1:500), GAPDH (D16H11) XP^®^ Rabbit mAb (CST^®^, 5174, dilution: 1:1000), and HRP-conjugated secondary antibody: HRP-Goat Anti-Rabbit IgG (Proteintech^®^, Rosemont, IL, USA, 00001-2, dilution: 1:2000).

The bands were visualized using the Clarity™ ECL Western blotting Substrate chemiluminescence detection kit (Bio-Rad, Hercules, CA, USA, 170-5060) and the VWR™ Imager Chemi Premium gel documentation system with VWR™ Image Capture Software (version: 1.6.1.0, Radnor, PA, USA). For densitometry analysis, Western blot data were acquired using ImageJ software (version: 1.53k) bundled with 64-bit Java 1.8.0_172.

### 4.9. Statistical Analyses

All statistical analyses (one-way, factorial ANOVA or nonparametric Kruskal–Wallis ANOVA & Median Test) were carried out using TIBCO^®^ Statistica™ program (version: 14.4.0.15). *p* Values were calculated with Dunnett’s test (after One-way or factorial ANOVA) or multiple comparisons (after the Kruskal–Wallis test). Data are presented as average ± SD from at least 3 independent experiments.

## 5. Conclusions

In this study, we aimed for the further elucidation of the mechanisms of action of the cancer cytotoxicity of Ph-Asc, CQ, and resveratrol. The observations that both Ph-Asc and resveratrol caused DSBs in DNA [8] surfaced the idea that the induction of DSBs can be the mysterious common point that leads to the death of cancer cells. Three compounds with oxidative stress-generating capabilities were chosen to confirm this hypothesis. By comparing the effects of the three previously investigated compounds (Ph-Asc, RES, CQ) and the three currently investigated compounds (menadione, RSL3, H_2_O_2_) on DNA damage, bioenergetic status, ROS, and lipid ROS generation, it could be assessed that

1. The induction of DSBs is certainly a common point of their mechanism of action;
2. The observed cancer cell death due to the investigated treatments are independent of the bioenergetics status;
3. Contrary to the other investigated compounds, the DNA damaging effect of CQ seemed to be ROS-independent;
4. The well-known ferroptosis inducer RSL3 was unable to induce lipid peroxidation in the PDAC Mia PaCa-2 cell line. At the same time, it induced DSBs in the DNA and the RSL3-induced cell death could not be suspended by the well-known ferroptosis inhibitors. All these observations suggest the ferroptosis resistance of this cell line.

At the same time, the observed DNA damaging effect of RSL3 creates a new perspective.

## Data Availability

The data that support the findings of this study are available from the corresponding author upon reasonable request.

## Supplementary Materials

The following supporting information can be downloaded at: https://www.mdpi.com/article/10.3390/molecules30051031/s1, Figure S1: The PARP1 inhibitor, Olaparib did not affect the cell death induced by Ph-Asc, CQ or RES in MIA PaCa-2 cells; Figure S2: The comparison of the effect of inorganic Fenton reagent and H_2_O_2_ alone in MIA PaCa-2 cells; Figure S3: The oxidative aspects of menadione induced cell death in HT-1080 cells.
