# Supplementary material for: Research for a Common Thread: Insights into the Mechanisms of Six Potential Anticancer Agents
Source: Molecules. 2025 Feb 24;30(5):1031. doi: 10.3390/molecules30051031 (PMC11901853; doi:10.3390/molecules30051031)
Supplement: Supplementary file 1 [file molecules-30-01031-s001.zip › molecules-3422172-supplementary.pdf]

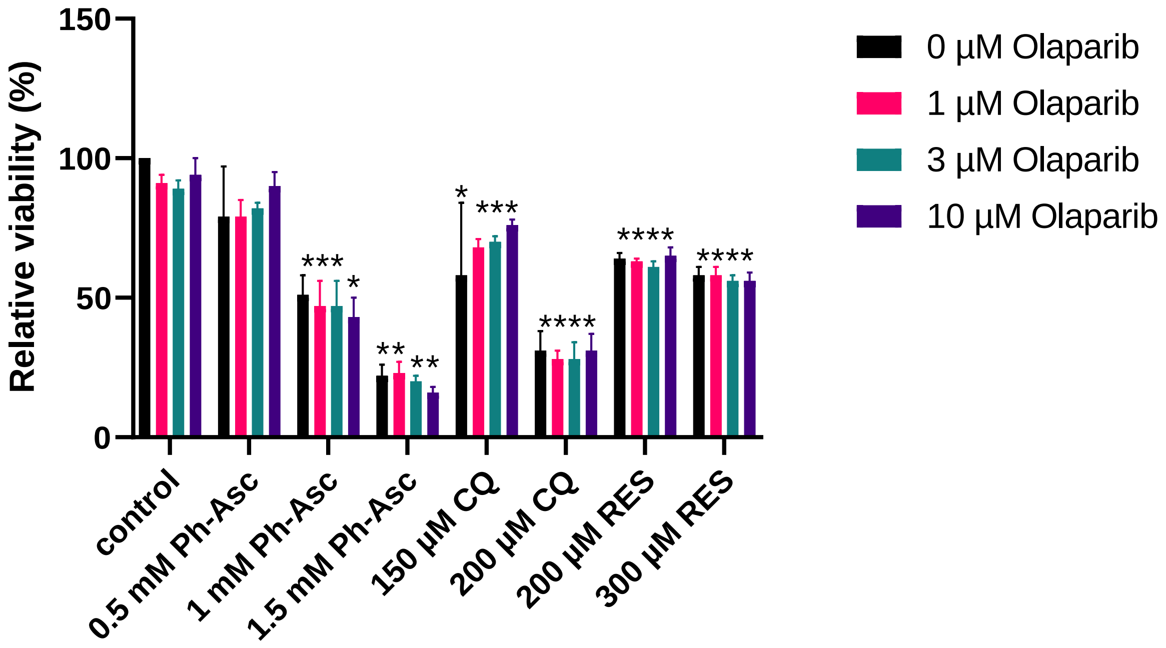

Figure S1. The PARP1 inhibitor, Olaparib did not affect the cell death induced by Ph-Asc, CQ or RES in MIA PaCa-2 cells

*MIA PaCa-2 cells were cultured on 96 well plates for 24 hours and then treated with the indicated compounds and concentrations (0.5-1.5 mM Ph-Asc, 150 µM and 200 µM CQ, and 200 µM and 300 µM RES) in the presence or absence of Olaparib (1-10 µM). Cell viability was measured by the MTT assay as described below. Data are normalized to untreated control and each data point represents the average ± SD from at least 3 independent experiments. *significantly different (p<0,05) from untreated control and #significantly different (p<0,05) from group control (same compound and concentration without Olaparib co-treatment).*

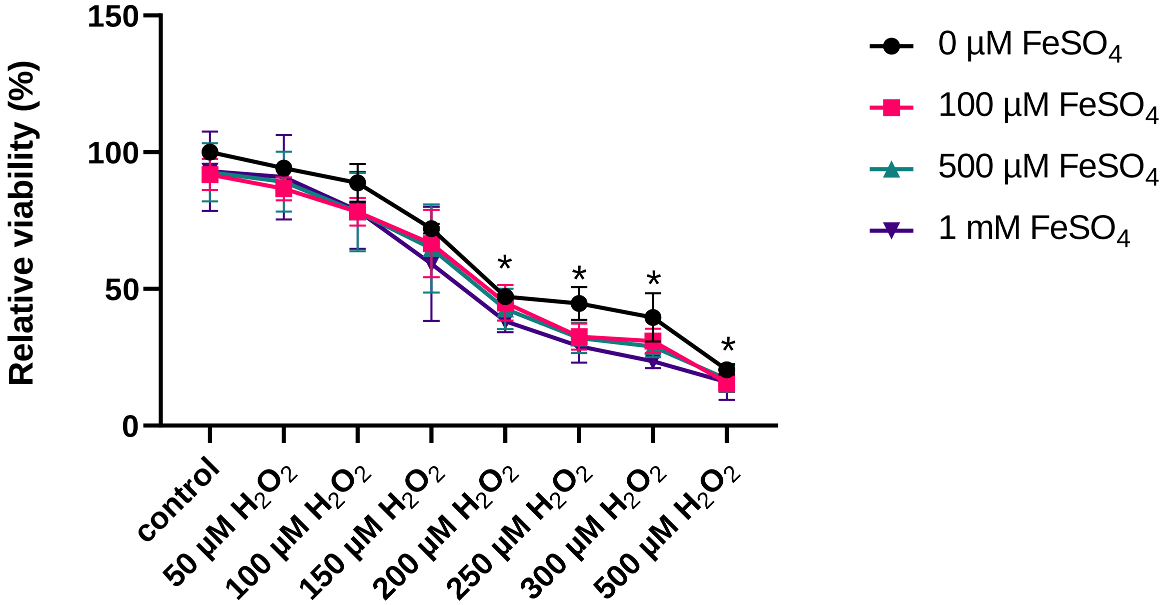

Figure S2. The comparison of the effect of inorganic Fenton reagent and H_2O_2 alone in MIA PaCa-2 cells

*MIA PaCa-2 cells were cultured on 96 well plates for 24 hours and then treated with the indicated concentrations of $FeSO_4$ (100 µM – 1 mM) for 1 hour, after the cells were washed twice with PBS and treated with the indicated concentrations of H_2O_2 (50-500 µM). Cell viability was measured by the MTT assay as described below. Data are normalized to untreated control and each data point represents the average ± SD from at least 3 independent experiments. *significantly different (p<0,05) from untreated control and #significantly different (p<0,05) from group control (same H_2O_2 concentration without $FeSO_4$ co-treatment).*

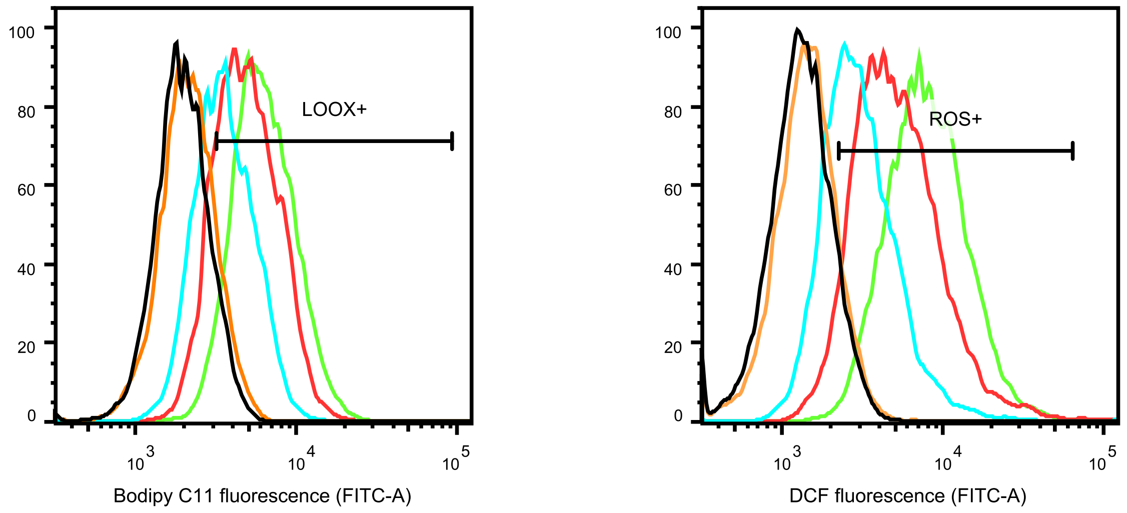

	% of total cells in LOOX+
control	10.4
10 µM menadione	14.9
20 µM menadione	60.2
30 µM menadione	82.4
50 µM menadione	95.3

	% of total cells in ROS+
control	10.6
10 µM menadione	13.3
20 µM menadione	71.0
30 µM menadione	93.7
50 µM menadione	99.1

Figure S3. The oxidative aspects of menadione induced cell death in HT-1080 cells.

HT-1080 cells were cultured on 24 well plates for 24 hours, pre-treated with the indicated fluorescent probe (H2DCFDA Invitrogen™ for ROS and BODIPY™ 581/591 C11 for lipid peroxidation measurement) for 30 minutes and then treated for 1 hour with the indicated concentratoins of menadione (10-50 µM). Cells were prepared, stained and measured with a flow cytometer as described below. Control samples were fluorescently labeled but untreated. One representative of at least three experiments is shown. Cell population was gated using SSC-FSC and propidium iodide negative staining for singlet, living cells.
